# Analysis of the protein network of cholesterol homeostasis maintenance in a mouse model of Alzheimer’s disease

**DOI:** 10.1186/1750-1326-8-S1-P37

**Published:** 2013-09-13

**Authors:** Marco Segatto, Pamela Rosso, Sandra Moreno, Valentina Pallottini

**Affiliations:** 1 Deapartment of Science, University Roma Tre, Rome, Italy

## Background

Cholesterol plays essential roles in the physiology of the central nervous system, ranging from axonal transport to synaptic plasticity [1,2]. Cholesterol metabolism maintenance is guaranteed by an intricate regulatory protein network, and imbalances in this fragile homeostatic regulation can easily lead to neurodegenerative disorders [3]. Molecular genetic evidence on apolipoprotein E isoforms has implications for cholesterol in the onset of Alzheimer’s disease (AD) [4]. Moreover, a plethora of data suggest that statins, HMG CoA reductase (HMGR) inhibitors widely used in therapies against hypercholesterolemia, could reduce the risk of developing AD [5]. Despite this evidence, no systematic research is being done to study the putative deregulation of cholesterol homeostasis in AD. Here, we analyzed the main proteins involved in cholesterol homeostasis maintenance in Tg2576 mouse model of AD.

## Materials and methods

Activation state and protein expression of HMGR, LRP1, LDLr and SRB1 were evaluated by Western blot both in hippocampus and brain cortex of Tg2576 transgenic mice. Neuronal and/or astrocytic protein localization was analyzed by immunofluorescence double staining.

## Results

No alterations were detectable in the activation state and protein level expression of HMGR, the key enzyme committed to cholesterol biosynthesis. By contrast, the expression of lipoprotein receptors LDLr, LRP1 and SRB1 were strongly increased in the hippocampus of Tg2576 if compared to age-matched wild type mice.

## Conclusions

The present work shows profound and marked alterations in the main proteins involved in lipoprotein metabolism, suggesting that local cholesterol transport could be impaired in a mouse model of AD. Whether these modifications could be the cause or the consequence of the disease still remains to be elucidated. A better comprehension of the molecular basis linking cholesterol metabolism and AD could be of importance when designing novel therapeutic strategies for neurodegenerative pathologies.
